# Domestication Shapes the Community Structure and Functional Metagenomic Content of the Yak Fecal Microbiota

**DOI:** 10.3389/fmicb.2021.594075

**Published:** 2021-03-31

**Authors:** Haibo Fu, Liangzhi Zhang, Chao Fan, Chuanfa Liu, Wenjing Li, Jiye Li, Xinquan Zhao, Shangang Jia, Yanming Zhang

**Affiliations:** ^1^Key Laboratory of Adaptation and Evolution of Plateau Biota, Northwest Institute of Plateau Biology, Chinese Academy of Sciences, Xining, China; ^2^Qinghai Provincial Key Laboratory of Animal Ecological Genomics, Xining, China; ^3^University of Chinese Academy of Sciences, Beijing, China; ^4^Datong Yak Breeding Farm of Qinghai Province, Datong, China; ^5^College of Grassland Science and Technology, China Agricultural University, Beijing, China

**Keywords:** Yak, domestication, 16S rDNA, shotgun metagenomic sequencing, fecal microbiota

## Abstract

Domestication is a key factor of genetic variation; however, the mechanism by which domestication alters gut microbiota is poorly understood. Here, to explore the variation in the structure, function, rapidly evolved genes (REGs), and enzyme profiles of cellulase and hemicellulose in fecal microbiota, we studied the fecal microbiota in wild, half-blood, and domestic yaks based on 16S rDNA sequencing, shotgun-metagenomic sequencing, and the measurement of short-chain-fatty-acids (SCFAs) concentration. Results indicated that wild and half-blood yaks harbored an increased abundance of the phylum *Firmicutes* and reduced abundance of the genus *Akkermansia*, which are both associated with efficient energy harvesting. The gut microbial diversity decreased in domestic yaks. The results of the shotgun-metagenomic sequencing showed that the wild yak harbored an increased abundance of microbial pathways that play crucial roles in digestion and growth of the host, whereas the domestic yak harbored an increased abundance of methane-metabolism-related pathways. Wild yaks had enriched amounts of REGs in energy and carbohydrate metabolism pathways, and possessed a significantly increased abundance of cellulases and endohemicellulases in the glycoside hydrolase family compared to domestic yaks. The concentrations of acetic, propionic, n-butyric, i-butyric, n-valeric, and i-valeric acid were highest in wild yaks. Our study displayed the domestic effect on the phenotype of composition, function in gut microbiota, and SCFAs associated with gut microbiota, which had a closely association with the growth performance of the livestock. These findings may enlighten the researchers to construct more links between economic characteristics and gut microbiota, and develop new commercial strains in livestock based on the biotechnology of gut microbiota.

## Introduction

Wildlife domestication is one of the most important events in the last 10,000 – 15,000 years of human history (Wendorf and Schild, 1998). Many types of wildlife, including insects, birds, and mammals, have been domesticated by humans to provide ample food and clothing, support large population sizes, and develop the expanse of civilization (Diamond, 2002; Boyazoglu et al., 2005). Artificial selection modifies not only the phenotypes of domestic animals, such as color, fur, body size, and personality, but also the genotypes, causing species divergence or the emergence of new species (Robison et al., 2006; Qiu et al., 2015; Pendleton et al., 2018). The gut microbiome, as the second genome of organisms, plays a crucial role in the growth and development of individuals (Grice and Segre, 2012; Shi et al., 2014). Recently, with developments in metagenome and hologenome sequencing, studies have reported the interdependence between the host and their symbionts, and that the microbial community aids the host in adapting to the challenges of varying environments (Mendoza et al., 2018). The gut microbiomes of mammals provide vital functions for their hosts, such as training the immune system throughout life, metabolism, and the biosynthesis of vitamins. This crucial relationship between mammals and their gut microbiota is the result of long-term coevolution (McFall-Ngai et al., 2013; Zeder, 2015; Alessandri et al., 2019). Although gut microbiota is a major research topic in microbial ecology, the effects of domestication on the gut microbiome of herbivorous mammals is still far from being fully understood (Metcalf et al., 2017). During wildlife domestication, gut microbes are influenced by diet (Mcknite et al., 2012), environment (Nicholson et al., 2012; Chevalier et al., 2015), and artificial breeding (Leamy et al., 2014). Anthropogenic forces may have reshaped the mammalian gut microbial composition and its subsequent metabolism, because natural habitats and host genetics are often greatly revised by such forces. However, the gut microbiota of domesticated species is also influenced by the current climate and vegetation (Chen C.Y. et al., 2018). Thus, it is difficult to distinguish the combined effects of genetics and ecological environments on the gut microbiota. Compared to readily digestible starch, fat and protein for most mammals, the cellulose and hemicellulose are indigestible substances (Gomez et al., 2015). Even in ruminants, it is difficult to digest completely in rumen for these indigestible substances (Lippke et al., 1986). As the auxiliary digestive organ of ruminants, the hindgut plays an important role in the utilization of indigestible substances, such as hemicellulose and pectin, which, largely, can reflect the influence of domestication on the utilization efficiency of food resources (Faichney, 1969; Hoover, 1978). Those individuals, who harbored strong capacity of digest indigestible substances in hindgut, may obtain more ecological advantages during utilization of food resources.

Yaks, a keystone species of the Qinghai Tibetan Plateau (QTP), are widely distributed on the QTP, numbering more than 14 million. They were domesticated by Tibetan people between 6,000 and 12,000 years ago (Guo et al., 2006; Wang et al., 2010). During the domestication of yaks, desirable traits, such as certain colors, fur types, and tameness, were chosen and strengthened by humans to breed appropriate yaks that satisfied anthropic needs for survival in the QTP (Qiu et al., 2015). Some studies reported that the phenotypic and behavioral characteristics of domestic yaks (*Bos grunniens*) are markedly different from those of their wild counter parts, wild yaks (*Bos mutus*), with changes in genetic variations and dwelling environments (Andersson and Georges, 2004; Rubin et al., 2010; Vigne, 2011; Larson and Burger, 2013). Geneticists have confirmed, that the host genes linked to specific phenotypes are advanced under selection, such as genes improving tameness, and a reduction in the copy number of sugar metabolism genes in the domestic yak (Qiu et al., 2015; Zhang X. et al., 2016). However, little is known about the gut microbiota under artificial selection.

The Qinghai Wild Yak Rescue Center was founded in 1,952, it is an organization that specializes in the rescue of wild yaks injured in the field, and in yak breeding by hybridization based on wild and domestic individuals, where wild yaks (*Bos mutus*), half-blood and domestic yaks (*Bos grunniens*) share the same environment. It provides ample opportunities to decouple the effects of host domestication.

To explore how host genetics reshaped the mammalian gut microbiota, we collected fecal samples from the Qinghai Wild Yak Rescue Center and analyzed the compositional and functional variations in fecal microbiota using 16S rDNA sequencing. Moreover, shotgun metagenomics based on the fecal samples from the three types of yaks were used to measure the variation in Kyoto Encyclopedia of Genes and Genomes (KEGG) pathways, rapidly evolved genes (REGs), and enzyme profiles of cellulase and hemicellulase. The results were further confirmed by surveying the short-chain fatty acid (SCFA) concentration yielded by the fecal microbiota. We focused on the following questions: (1) whether host genetic variation of domesticated mammals caused by anthropogenic forces could dramatically reshape the fecal microbiomes; (2) how domestication changes crucial metabolic pathways in bacteria that may be linked to nutrition metabolism; and (3) whether modifications in host genetics results in distinct profiles of the glycoside hydrolase (GH) family of cellulose and hemicellulose enzymes in the fecal microbiota of domesticated mammals, compared to their wild conspecifics.

Here, we investigated the variations in microbial community structure and function of wild, domesticated, and half-blood yaks, exposed to the same environmental conditions. We also examined the changes in the REGs and enzyme profiles of GH caused by domestication. Our results may assist to better understand the evolutionary associations between the host and their gut microbiota in herbivorous mammals under artificial selection.

## Materials and Methods

### Animal Material and Sample Information

We collected fresh feces from three types of yaks (domestic, half-blood, and wild) at the Datong Yak Breeding Farm in Qinghai Province (37°15′N, 101°22.8′E, and 2,980 m above sea level). More than 60 offspring of wild yaks that were injured by wolves (*Canis lupus Linnaeus*) or brown bears (*Ursus arctos*) were rescued by workers of the Datong Breeding Farm in Hoh Xil Region since the 1980s. Domestic yaks belonged to local individuals, and the half-blooded yaks are the F1 generation of male wild yaks and female domestic yaks (Jiang and Zhonglin, 2005).

The wild and half-blood yaks are raised on a small hillside of the farm, about 300 hectares, surrounded by a 2 m high iron fence to prevent them from escaping. We hid in the shelter, guided by local workers, and observed the excretion of feces of the target animals. After determining the identity of the wild or half-blood yak, we collected the samples within two hours. Samples of domestic yaks were taken within a 1 km radius of the breeding center. Under the guidance of farm workers, we followed the yaks, collected the samples, and stored them in liquid nitrogen.

Fresh fecal samples of 29 healthy adult yaks, including 10 wild yaks, 11 half-blood yaks, and eight domestic yaks were collected for 16S rDNA in September 2017. In addition, nine fecal samples were prepared for metagenome sequencing, each group contained three samples. All samples were immediately frozen in cryotubes in liquid nitrogen. Then, the samples were stored at −80°C for further analysis. In addition, we listed the nutrition composition of diet for yaks in Supplementary Table 1. We performed the animal experiments following the Administration of Laboratory Animals established by the Ministry of Science and Technology of the People’s Republic of China.

### DNA Extraction and 16S rDNA Gene Amplification Sequencing

We carried out the extraction and amplification of 16S rRNA gene based on the previous study (Fu et al., 2020). The amplification was conducted targeting the V3 and V4 regions of 16S rRNA gene using the primers 341F (5′-CCTAYGGGRBGCASCAG-3′) and 806R (5′-GGACTACNNGGGTATCTAAT-3′) (Claesson et al., 2010; Vilo and Dong, 2012). The quantification and purification of the polymerase chain reaction (PCR) products according to the previous study (Fu et al., 2020). To ensure that there was no contamination, a positive and negative control was used during PCR. Then, the sequencing libraries were prepared and measured following the method in previous study (Fu et al., 2020). Finally, the libraries were sequenced on the HiSeq 2,500 platform of Illumina (Illumina Inc., San Diego, CA, United States) to produce 250 bp paired-end reads.

We obtained the gut metagenomic DNA from nine fecal samples (three for each type of yak, respectively) according to the previous method (Fu et al., 2020). After that, we determined the DNA concentration using the proved method in other study (Fu et al., 2020).

### Shotgun Metagenomic Sequencing

Qualifying DNA samples were randomly interrupted using a Covaris ultrasonic crusher and produced approximately 150 bp libraries (Bowers et al., 2015). The whole libraries were prepared using the steps of end repair with a 3′ A tail, ligation of adapters and purification (Bentley et al., 2008). After that, library quality was assessed and sequenced with 150 bp paired-end reads based on the previous methods (Tringe and Rubin, 2005; Fu et al., 2020). Finally, we obtained an average of 540 million metagenomic-jointed reads (12 Gb) per sample from the sequencing platform (Edgar, 2004).

### OTU Clustering and Species Annotation

Raw forward-read sequences were analyzed using the quantitative insights into microbial ecology (QIIME) pipeline (Version 1.9.1) (Caporaso et al., 2010). Sequence analysis was performed with Uparse software (Uparse v7.0.1001^1^) (Edgar, 2013), and preprocessed sequences were conducted at a 97% cut-off of nucleotide sequence similarity level. Taxonomic information was annotated to the OTUs based on the Greengenes 13_8 reference database^2^ (DeSantis et al., 2006). The sequences clustered into mitochondria and chloroplast were removed from the OTU table. The unclassified sequences at the Kindom level were discarded. A standard sequence number corresponding to the least sequences (89,510) of the samples was adopted to normalize the OTU abundance information.

### Treatment of the Raw Metagenomic Data

We measured the quality of the raw data using the fastqc_v0.11.5, then filtered the low quality sequences using Trimmomatic v0.36 with the parameters of LEADING:20 TRAILING:20 SLIDINGWINDOW:3:15 MINLEN:140. After that, we measured the quality of sequences filtered by Trimmomatic v0.36 again using fastqc_v0.11.5 to ensure the quality of filtered sequences for the further analysis. Owing to the potential containment of host sequences in the metagenomic data, we searched against the host database using SoapAligner (soap2.21,^3^) based on the filtered data, and the parameters are as follows: identity ≥90%, −l 30, −v 7, −M 4, −m 200, and −x 400 (Law et al., 2013).

### Glycoside Hydrolase Families Responsible for Cellulose and Hemicellulose Degradation

We predicted the open reading frames (ORFs) based on the trimmed metagenomic reads using the FragGeneScan v1.15 (Rho et al., 2010). Then, we searched against the complete, non-redundant sequences in the dbCAN database^4^ using the basic local alignment search tool (BLASTp) best hits with a cut-off E-value of 1e-5 based on the predicted ORFs (Yin et al., 2012), the ORFs for candidate proteins that have sequence homologous with GH families were extracted (Yin et al., 2012). Next, all the clean DNA reads corresponding to GH families were aligned against the bacterial genome database for species assignment^5^ using BLASTn. Lastly, a table with CAZy IDs and species assignments was made by running local perl scripts written by ourselves.

### Gene Set Enrichment Analysis Based on the Metagenomic Data

Gene set Enrichment Analysis (GSEA) was performed to identify differential abundance of microbial gene pathways using the KEGG database. Microbial genes from all samples were represented as KOs, and a *p*-value was generated for each KO using either a gamma model or a mixture model (Schwimmer et al., 2019). The relative enrichment of KEGG pathways within KO rankings was calculated using the R piano package (Varemo et al., 2013). Positive and negative gene-level statistics belonging to a gene set were separated based on the direction of the normalized Wilcoxon signed-rank test statistic, and for each of the two subsets, the absolute sum was divided by the total number of genes in the set (Katoh and Standley, 2013). Significance was determined at *p* < 0.05 after FDR correction (Varemo et al., 2013; Schwimmer et al., 2019).

### Calculation of One-to-One Orthologs and Identification of Rapidly Evolving Genes Based on the *Ka/Ks* in the Gut Microbiota

For any two types of yaks (a, b), the one-to-one orthologs in the gut microbiota were defined as homologous genes. Then, we conducted a two-way microbial protein alignment to identify the homologous genes using the BLAST software (*E*-value threshold was 1e-5) (Hulsen et al., 2006). Then, we carried out a microbial protein multiple sequence alignment using the software multiple alignment by fast Fourier transform (MAFFT) v7.402 with the default parameters (Katoh and Standley, 2013). The corresponding aligned DNA sequences were obtained using the pal2nal.pl script (version V4,^6^). Subsequently, non-synonymous-to-synonymous substitution rate (*Ka/Ks* ratio) was calculated using the pairwise model in the software phylogenetic analysis by maximum likelihood (PAML) v8.0 (Yang, 2007; Katoh and Standley, 2013). *Ka*/*Ks* < 1 indicates purifying selection, whereas *Ka*/*Ks* > 1 is the signature of positive selection (Hurst, 2002). Based on this definition, those identified orthologs with *Ka*/*Ks* > 1 (*FDR*-corrected *p* < 0.05) were considered as REGs (domestic yak vs. wild yak: 18,216 REGs with 191,852 orthologs; domestic yak vs. half-blood yak: 18,831 REGs with 194,930 orthologs; half-blood yak vs. wild yak: 19,765 REGs with 202,590 orthologs). Based on the KEGG annotation of homologous microbial genes results, we identified the enriched KEGG of accelerating evolution genes using Fisher’s exact test in R. The formula was as follows: fisher.test {matrix [c (A, B, C, D), nc = 2], alternative = “two. Sided,” conf.level = 0.99, simulate. *P*-value = false, *B* = 10000}. In the formula, A represents the number of rapidly evolving genes in a specific pathway; B represents the number of non-rapidly evolving-genes in a specific pathway; C represents the number of all homologous genes in a specific pathway; D represents the number of homologous genes in a non-specific pathway. The statistical threshold of multiple testing using the FDR corrections was *p* < 0.05.

### Determination of SCFAs Concentration

The short-chain fatty acids in yak feces were determined using propyl chloroformate (PCF) derivatization followed by gas chromatography-mass spectrometry (GC-MS) (Zheng et al., 2013). The experimental procedures, instrument and reagent used during the operation were based on the previous methods (Fan et al., 2020).

### Bioinformatics and Statistical Analyses

The taxa with significant differences between different types of yaks were measured using a Kruskal-Wallis test followed by Dunn’s *post hoc* multiple-comparison test in GraphPad Prism v7.00. Alpha diversity (including Shannon-Wiener and Simpson) was calculated using Python scripts in QIIME (Version 1.9.1) in workflows *Alpha_rarefaction.py* (Shannon, 1948; Simpson, 1949; Chao, 1984; Chao and Lee, 1992; Lozupone et al., 2011; Parfrey et al., 2014), finally the alpha diversity was visualized in GraphPad Prism v7.00, and measured the differences using a Kruskal-Wallis test followed by Dunn’s *post hoc* multiple-comparison test. The beta diversities were tested using permutational analysis of variance (PERMANOVA) with 999 permutations in R 3.2.2, using the function *adonis*. The significant level in this study was determined according to the common standard (*p* > 0.05, no significance; *p* < 0.05, ^∗^; *p* < 0.01, ^∗∗^; *p* < 0.001, ^∗∗∗^). LEfSe was used to identify the different bacterial taxa among different groups using a common standard (*LDA* scores > 3, *p* < 0.05).

The read numbers of GH families associated with the cellulases and hemicellulases were visualized and measured the differences using the Welch *t*-test (*p* < 0.05) in GraphPad Prism v7.00. The SCFA results were analyzed using the Wilcoxon rank-sum test (*p* < 0.05).

### Data Availability

The 16S rDNA as well as the whole-metagenome data in this study can be freely retrieved from the NCBI Sequence Read Archive with project accession Nos. PRJNA528194 and PRJNA529943, respectively.

## Results

### Domestication Modified the Diversity and Community Structure of the Yak Fecal Microbiota

**FIGURE 1:**
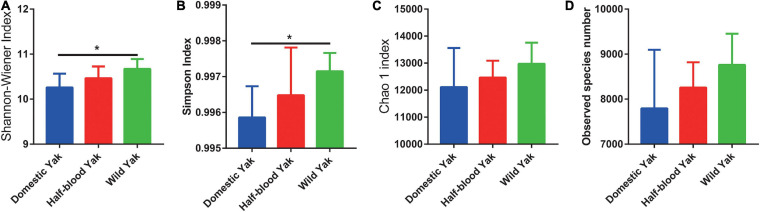
The alpha diversity of gut microbial communities in three types of yaks, asterisks indicate significance level between groups (*p* > 0.05, no significance; *p* < 0.05, *; *p* < 0.01, **; *p* < 0.001, ***). **(A)** The Chao 1 index **(B)** and observed species number. **(C)** The Shannon-Wiener, **(D)** and Simpson indices.

After sequences were subjected to quality filtering and assembly, 3,065,986 16S rRNA gene sequences were obtained. The composition of the fecal microbiota in yaks was dominated by the phyla *Firmicutes*, *Bacteroidetes*, *Verrucomicrobia*, *Proteobacteria*, and *Actinobacteria* (Supplementary Table 2). The results of alpha diversity showed that the Shannon-Wiener and Simpson indices were significantly higher in wild yaks than in domestic yaks (Shannon-Wiener: χ^2^ = 8.099, df = 2, *p* = 0.0149; Simpson: χ^2^ = 7.369, df = 2, *p* = 0.0141) (Figures 1A,B). However, there were no significant differences between the three types of yaks in both the Chao 1 index and observed species number (Chao 1: χ^2^ = 2.890, df = 2, *p* = 0.2358; Observed species number: χ^2^ = 4.411, df = 2, *p* = 0.1102) (Figures 1C,D).

### Characteristics of Fecal Microbial Composition in Different Types of Yaks

**FIGURE 2:**
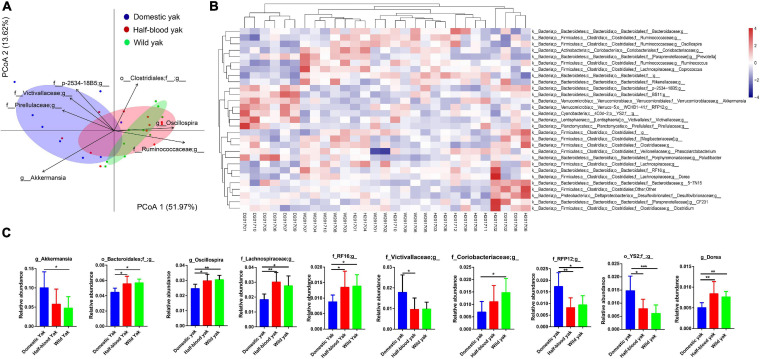
Composition of fecal microbiota of the three types of yaks at the genus level, the letters “D,” “H,” and “W” represent domestic, half-blood, and wild yaks, respectively. **(A)** Bray-Curtis dissimilarity of gut microbial communities among the three types of yaks based on top 30 genera, ellipses with 95% confidence interval around the centroid of each group are displayed in PCoA, the genera which have significant correlation with the ordination in PCoA are displayed using the arrows (permutation test, *p* < 0.01), with the length of the arrow representing the goodness of fit statistic, squared correlation coefficient. **(B)** Heatmap of the top 30 genera based on the relative abundance of gut mcirobiota among the three types of yak, complete linkage clustering was used. **(C)** Relative abundance of the gut microbiota indicating the significantly discrepancies among the three types of yak at the genus level across 29 samples, asterisks indicate significance level between groups (*p* > 0.05, no significance; *p* < 0.05, *; *p* < 0.01, **; *p* < 0.001, ***).

We used the principal-coordinate analysis (PCoA) based on the Bray-Curtis distance of the microbial community at the genus and phylum level to investigate the similarity of the gut microbial community structure for each individual between the three types of yak. We found that clear clustering of samples by host family (Figure 2A and Supplementary Figure 1A), and PERMANOVA, based on Bray-Curtis distance, revealed that significant differences among the three groups were observed at the genus and phylum level (genus: *F*_2_,_26_ = 4.3, *R*^2^ = 0.25, *p* = 0.002, permutations = 999; phylum: *F*_2_,_26_ = 6.3, *R*^2^ = 0.32, *p* = 0.001, permutations = 999) (Figure 2A and Supplementary Figure 1A). The distribution of individuals in the PCoA was mainly driven by dominant taxon at both genus and phylum level (Figure 2A and Supplementary Figure 1A). *Akkermansia*, family Pirellulaceae unclassified genus, family Victivallaceae unclassified genus, and family p-2534-18B5 unclassified genus were positively correlated and contributed significantly to the domestic group, while genus *Oscillospira*, family Ruminococcaceae unclassified genus were positively correlated and contributed significantly to the wild yaks. Furthermore, the heatmap of the relative abundance of the genera with the cluster of the groups shows that *Akkermansia*, family Pirellulaceae unclassified genus, family Victivallaceae unclassified genus, and family p-2534-18B5 unclassified genus, which were positively correlated to the domestic group, form a cluster in the hierarchical cluster; while genus *Oscillospira*, family Ruminococcaceae unclassified genus, which were positively correlated to wild yaks, form another cluster in the hierarchical cluster (Figure 2B). At the phylum level, the phylum *Verrucomicrobia* and Lentisphaerae were positively correlated and contributed significantly to the domestic yaks (Supplementary Figure 1A), and form a cluster in the hierarchical cluster of the heatmap (Supplementary Figure 1B); while the phylum *Firmicutes* was positively correlated and contributed significantly to the half-blood and wild yaks (Supplementary Figure 1A), and forms another cluster in the hierarchical cluster of the heatmap (Supplementary Figure 1B). Moreover, three types of yak shared 242 (68.9%) genera; the shared genus number between domestic and wild yak is 257 (73.2%), while the shared genus number between half-blood and wild yak is 290 (82.6%). At the phylum level, among 22 phyla, three types of yak share 19 phyla (86.4%), and the wild yaks have two unique phyla (9.1%), though two unique phyla (Thermi and SR1) are rare in the wild yaks (Supplementary Table 2). We further identified the significantly different genus based on the top 30 genera, as they spanned over 95% of the total microbial community. We found that genus *Akkermansia*, family Victivallaceae unclassified genus, family RFP12 unclassified genus, order YS2 unclassified family displayed significantly higher abundance in domestic yaks (Figure 2C and Supplementary Table 3), and the order Bacteroidales unclassified family, genus *Oscillospira*, family *Lachnospiraceae* unclassified genus, family RF16 unclassified genus, family *Coriobacteriaceae* unclassified genus, and genus *Dorea* displayed significantly higher abundance in wild yaks (Figure 2C and Supplementary Table 3). At the phylum level, the abundance of phylum *Verrucomicrobia*, Lentisphaerae, and *Cyanobacteria* are significantly higher in domestic yaks (Supplementary Figure 1C and Supplementary Table 3). The abundance of phylum *Firmicutes*, *Actinobacteria*, TM7, and Elusimicrobia are significantly higher in wild yaks (Supplementary Figure 1C and Supplementary Table 3).

**FIGURE 3:**
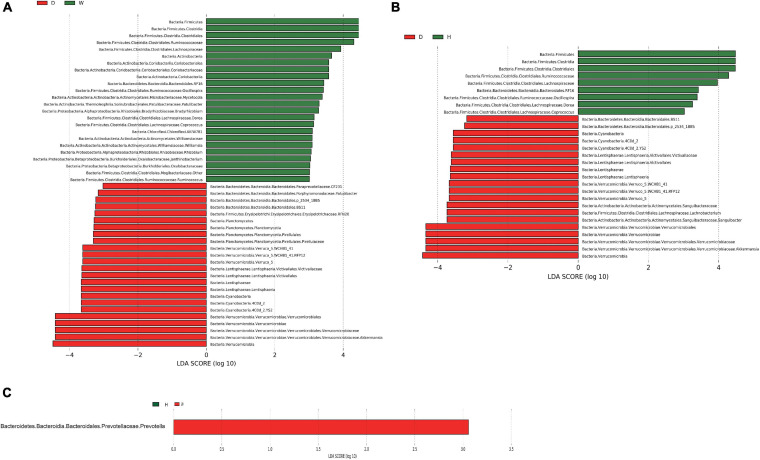
The linear discriminant analysis effect size (LEfSe) shows the significantly different taxa of the gut microbiota between the different groups at the genus level (*LDA* scores > 3.0, *p* < 0.05); the letters “D,” “H,” and “W” represent domestic, half-blood, and wild yaks, respectively. **(A)** The significantly different taxa between domestic and wild yaks. **(B)** The significantly different taxa between domestic and half-blood yaks. **(C)** The significantly different taxon between half-blood and wild yaks.

To further illustrate the influence of domestication on the bacteria in yaks, we conducted a linear discriminant analysis (LDA) effect size (LEfSe) analysis based on the genus and phylum level for the different taxon with lineage classification (*LDA* scores > 3.0, *p* < 0.05) (Figures 3A–C and Supplementary Figures 2A,B). Domestic and wild yaks harbored the richest significantly different taxa (total: 48; wild > domestic: 24, domestic > wild: 24) (Figure 3A), domestic and half-blood yaks possessed a moderate number of significantly different taxa (total: 29; half-blood > domestic: 9, domestic > half-blood: 20) (Figure 3B). *Prevotella* was the only significantly different taxon between the half-blood and wild yaks (Figure 3C). At the phylum level, the *Firmicutes*, *Actinobacteria*, Elusimicrobia, and TM7 displayed a significantly higher abundance in wild yaks than in domestic yaks, and the *Verrucomicrobia*, Lentisphaerae, *Cyanobacteria*, *Planctomycetes*, and Tenericutes were significantly higher in domestic yaks than in wild yaks (*LDA* scores > 2.0, *p* < 0.05) (Supplementary Figure 2A). Moreover, the *Firmicutes*, TM7, and Spirochetes displayed significantly higher abundances in half-blood yaks than in domestic yaks, and the *Verrucomicrobia*, Lentisphaerae, and *Cyanobacteria* were significantly higher in domestic yaks than in half-blood yaks (*LDA* scores > 2.0, *p* < 0.05) (Supplementary Figure 2B). No significantly different taxon was identified between half-blood yaks and wild yaks (*LDA* scores > 2.0, *p* > 0.05).

### Gene Set Enrichment Analysis and REGs

Based on GSEA, 16 pathways were differentially abundant in one or more pairwise comparisons. The most striking differences were found in genes related to methane emission in domestic yaks (Table 1). GSEA also identified the insulin-signaling pathway, which contributed to the synthesis of glycogen, lipids, and proteins, as a highly enriched pathway with six upregulated KEGG orthologs (KOs) in the metagenome dataset from wild yaks (Table 1). Additionally, the mitogen-activated protein kinase (MAPK) signaling pathway associated with cell division was significantly enriched in wild yaks (Table 1).

**TABLE 1:** Metabolic pathways enriched in different types of yak.

	Enriched in Pathway	KEGG ID	KOs in pathway	KOs detected in study	GSEA statistic	*p*-value	*q*-value	KOs up in rank	KOs down in rank
Domestic yak	Photosynthesis	ko00195	63	21	0.743778489	0.00140056	0.058292697	19	2
	Ribosome biogenesis in eukaryotes	ko03008	82	20	0.701824916	0.002808989	0.058292697	15	5
	Methane metabolism	ko00680	167	118	0.555595404	0.001116071	0.058292697	104	14
	Microbial metabolism in diverse environments	ko01120	1057	465	0.322232731	0.006085193	0.083271058	366	99
Half-blood yak	Lipopolysaccharide biosynthesis	ko00540	40	35	0.540141042	0.001766784	0.022438541	29	6
	Bacterial secretion system	ko03070	74	52	0.537770079	0.001785714	0.022438541	41	11
Wild yak	Autophagy-animal	ko04140	100	11	0.811226568	0.005780347	0.073048687	5	6
	Tuberculosis	ko05152	131	10	0.769289429	0.005617978	0.073048687	5	5
	Autophagy-yeast	ko04138	77	16	0.757671878	0.007575758	0.073048687	9	7
	Insulin signaling pathway	ko04910	86	12	0.737158547	0.006289308	0.073048687	6	6
	Phagosome	ko04145	95	10	0.670552842	0.011235955	0.073048687	6	4
	Endocytosis	ko04144	178	11	0.668547332	0.005780347	0.073048687	6	5
	Spliceosome	ko03040	122	10	0.616286826	0.011235955	0.073048687	6	4
	Glucagon signaling pathway	ko04922	55	11	0.60784035	0.005780347	0.073048687	3	8
	Epstein-Barr virus infection	ko05169	164	17	0.592686653	0.007633588	0.073048687	9	8
	MAPK signaling pathway-yeast	ko04011	90	14	0.547305429	0.006802721	0.073048687	6	8

*Metabolic pathways enriched in different types of yaks, gene set enrichment analysis (GSEA) results. Through the comparison among domestic, half-blood and wild yak, the metabolic pathways enriched in different types of yak were revealed.*

Further, we obtained 191,852 one-to-one orthologs in the metagenomes of wild and domestic yaks, 194,930 for domestic and half-blood yaks, and 202,590 for wild and half-blood yaks. After FDR correction, 18,216 (9.5%), 18,831 (9.7%), and 19,765 (9.8%) REGs were detected, respectively (Table 2; *p* < 0.05, Fisher’s exact test).

**TABLE 2:** Functional categories with significant enrichment in rapidly evolved genes (REGs).

	Enriched in pathway	KEGG ID	REG in pathway	REG detected in study	*p*-value	One-to-one orthologues	Number of REGs	Enriched REGs (proportion)
Wild yak vs domestic yak	Pentose and glucuronate interconversions	ko00040	399	2	2.16E-13			
	Sphingolipid metabolism	ko00600	474	2	2.96E-16			
	Pantothenate and CoA biosynthesis	ko00770	331	4	4.82E-09			
	Nicotinate and nicotinamide metabolism	ko00760	459	2	1.49E-15			
	Sulfur metabolism	ko00920	212	2	0.00000131			
	Thiamine metabolism	ko00730	456	4	2.28E-13			
	Lipopolysaccharide biosynthesis	ko00540	164	2	0.0000787			
	Total					191,852	18,216	9.5%
Wild yak vs half-blood yak	Phenylalanine, tyrosine and tryptophan biosynthesis	ko00400	525	5	2.2E-15			
	Lipopolysaccharide biosynthesis	ko00540	182	3	0.0000769			
	Folate biosynthesis	ko00790	263	2	1.42E-08			
	Thiamine metabolism	ko00730	410	4	4.32E-12			
	Total					202,590	19,765	9.8%
Domestic yak vs half-blood yak	Monobactam biosynthesis	ko00261	228	2	0.000000288			
	One carbon pool by folate	ko00670	420	3	2.25E-13			
	Carbon fixation in photosynthetic organisms	ko00710	370	2	1.42E-12			
	Pantothenate and CoA biosynthesis	ko00770	347	5	7.66E-09			
	Porphyrin and chlorophyll metabolism	ko00860	245	2	6.33E-08			
	Drug metabolism – other enzymes	ko00983	225	4	0.0000184			
	Nicotinate and nicotinamide metabolism	ko00760	459	3	1.17E-14			
	Pentose and glucuronate interconversions	ko00040	372	2	1.48E-12			
	Biofilm formation – Escherichia coli	ko02026	270	2	9.92E-09			
	Folate biosynthesis	ko00790	227	2	0.000000287			
	Citrate cycle (TCA cycle)	ko00020	399	2	1.49E-13			
	Fructose and mannose metabolism	ko00051	490	4	7.66E-15			
	Total					194,930	18,831	9.7%

Enriched REGs among the wild, domestic, and half-blood yaks were significant in functional categories involved in energy, carbohydrate, and lipid metabolism, as well as glycan biosynthesis and metabolism (Table 2; *p* < 0.05, Fisher’s exact test). REGs of wild and domestic yaks were significantly involved in sulfur metabolism, pentose and glucuronate interconversions, sphingolipid metabolism, lipopolysaccharide biosynthesis, pantothenate and CoA biosynthesis, thiamine metabolism, and nicotinate and nicotinamide metabolism (Table 2; *p* < 0.05, Fisher’s exact test). Enriched REGs between half-blood and wild yaks were involved in lipopolysaccharide biosynthesis, folate biosynthesis, thiamine metabolism, and phenylalanine, tyrosine, and tryptophan biosynthesis (Table 2; *p* < 0.05, Fisher’s exact test).

### Profile of Enzymes Associated With Cellulose and Hemicellulose Degradation

**FIGURE 4:**
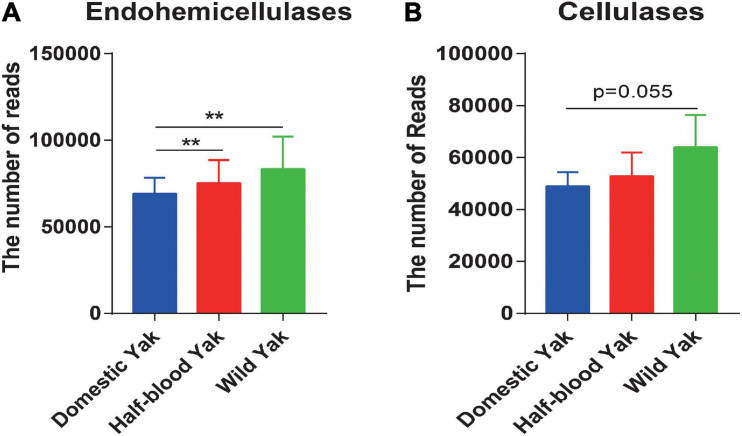
**(A,B)** The total read number of the GH families encoding hemicellulases and cellulases across the three types of yaks, asterisks indicate significance level between groups (*p* > 0.05, no significance; *p* < 0.05, *; *p* < 0.01, **; *p* < 0.001, ***).

We identified the GH family, which contains cellulases and hemicellulases, based on shotgun metagenome sequencing data. The results revealed that the total reads of GH families, which were responsible for hemicellulose degradation, were significantly higher in wild and half-blood yaks than in domestic yaks (Welch *t*-test, *p* < 0.05) (Figure 4A), and the total reads of GH families, which were responsible for cellulose degradation, were almost significant between wild and half-blood yak (Welch *t*-test, *p* = 0.055) (Figure 4B). To further investigate the relative contribution of bacteria encoding cellulases and hemicellulases, we choose GH10, GH28, and GH53 which degraded hemicellulose, and GH5 which degraded cellulose, and found that replacement or absence often occurred in the bacterial producer of the same enzyme (GH) between wild and domestic yaks (Supplementary Table 4).

### SCFA Concentrations

**FIGURE 5:**
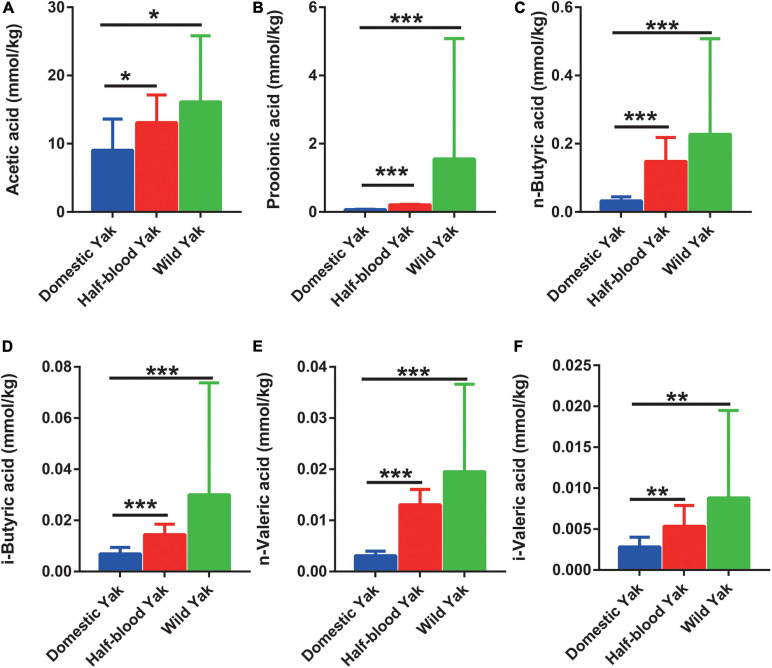
The SCFAs concentration of gut microbiota in the three types of yaks, asterisks indicate significance level between groups (*p* > 0.05, no significance; *p* < 0.05, *; *p* < 0.01, **; *p* < 0.001, ***). **(A)** Acetic acid, **(B)** propionic acid, **(C)** n-butyric acid, **(D)** i-butyric acid, **(E)** n-valeric acid, and **(F)** i-valeric acid.

We surveyed six dominant SCFAs, and the results showed that half-blood and wild yaks had significantly higher concentrations of acetic acid, propionic acid, n-butyric acid, i-butyric acid, n-valeric acid, and i-valeric acid than domestic yaks (Wilcoxon Rank-Sum test, *p* < 0.05) (Figures 5 A–F).

## Discussion

Domestication provides people important and stable food sources by modifying the genetic characters of wild animals, yet its influence on the gut microbiome is still poorly understood (Diamond, 2002; Boyazoglu et al., 2005). Here, we assessed the gut microbial community by comparing the wild, hybrid and domestic types of yaks in the same environment. We measured the diversity, difference of the gut microbial structure, and also explored the differences of metagenomic functions and SCFAs among three types of yaks. Most lineal wild ancestors of current domestic animals have been extinct (Hassanin and Ropiquet, 2004). However, we collected the wild, hybrid and domestic types of yaks at the same time, as the domestication of yaks is still in the early stage, and wild population still exists on QTP, though the population is small compared to the historical period (Shi et al., 2016). Thus, three types of yaks provided a rare opportunity to explore the domestic effects on the gut microbiota for us. We will discuss the domestic effects on the gut microbial structure, metagenomic functions, and SCFAs by comparing the three types of yaks in the following sections.

Recently, many studies have indicated that domestication decreases the diversity of the gut microbial community in horses, fruit fly larvae, and Eastern African cichlid fish, because of the loss of some taxa (Larson and Burger, 2013; Baldo et al., 2015; Deutscher et al., 2018). In this study, domestic yaks had a lower Shannon-Wiener index (Figure 1A), consistent with above results (Larson and Burger, 2013; Deutscher et al., 2018; Alessandri et al., 2019). Higher diversity of the gut microbiota is closely associated with adaptation to a diverse diet and expanding the breadth of the dietary niche of mammalian herbivores, which benefits the fitness of the host (Kohl et al., 2014; Li et al., 2019). Thus, higher Shannon-Wiener index of gut microbial community in wild yaks may imply that the wild individuals have evolved a broad dietary niche adaption to survive the harsh environment in the field.

Recently, there has been increasing evidence that the composition of gut microbiota in domesticated animals is substantially different from that of related wild species, such as domestic and wild horses, geese, and silkworms (Larson and Burger, 2013; Gao et al., 2016; Metcalf et al., 2017; Chen B. et al., 2018). Li et al., 2017 found that wild musk deer possess a higher abundance of the phylum *Firmicutes*, and a lower abundance of the genus *Akkermansia* than captive individuals. Additionally, wild house mice harbored an increased abundance of *Firmicutes* and reduced abundance of *Akkermansia* (Kreisinger et al., 2014). These two taxa of gut microbiota are strongly associated with the efficient harvest of energy from the diet (Nicholson et al., 2012). Furthermore, the study of the core gut microbiome in obese and lean twins suggested that the phylum *Actinobacteria* yielded 75% of obesity-enriched genes, while *Firmicutes* yielded the other 25% (Turnbaugh et al., 2009), suggesting that *Actinobacteria* and *Firmicutes* were closely associated with growth and fat deposition. In this study, the wild and half-blood yaks harbored a significantly increased abundance of *Firmicutes* and *Actinobacteria* and a reduced abundance of *Akkermansia* (Supplementary Figure 1C and Figure 2C), similarly to wild musk deer and wild house mice (Kreisinger et al., 2014; Li et al., 2017). These results may imply that an efficiency microbial biomarker for food resources utilization in wild yaks than in domestic yaks.

Methane metabolism enriched in domestic yak rather than wild and half-blood yak might imply that domestic yaks had a higher methane emission level, and further cause energy loss (Table 1). In fact, low-altitude ruminants, such as cattle and ordinary sheep, which experienced longer and stronger domestication than yak, possessed an increased methane emission phenotype in comparison to related high-altitude ruminants (yak and Tibetan sheep) (Zhang Z. et al., 2016). Additionally, the insulin-signaling pathway enriched in wild yaks might be a biomarker of assimilation (Table 1), as and may benefit growth by contributing to the repression of catabolism.

Adaptive divergence in the gene sequence may also contribute to the phenotypes of low-methane-yielding and high-SCFA organisms (Zhang Z. et al., 2016). The results showed the REGs of wild yaks were enriched in energy and carbohydrate metabolism pathways compared to domestic yaks, indicating that wild yaks potentially possessed the typical high-SCFA phenotype. The lower content of SCFAs in domestic yaks may further reflect changes in host biology compared to the wild yaks (Figures 5A–F). Conversely, the enrichment of REGs in domestic yaks showed that the pathways were associated with the methane emission pathway (Tables 1, 2). These results illustrated that artificial selection may cause rapid gene evolution of the gut microbiome and weaken the feed efficiency in domestic yaks.

Metagenomic sequencing further confirmed that the wild and half-blood yaks harbored more reads for endohemicellulases in GH families (Figure 4A). These findings implied that wild yaks might harbor a stronger capacity for fiber digestion, which might potentially promote the ability of the host to acquire more calories from the diet. Similar results show that wild individuals harbor an increased abundance of cellulose- or hemicellulose-degrading bacteria in wild house mice (Kreisinger et al., 2014). Furthermore, the wild saiga has a higher digestibility than the related domestic species in the same territory (Abaturov et al., 2003), and wild asses achieve higher digestibility than domesticated asses (Hummel et al., 2017). These common features occurring in different, unrelated, domesticated animals suggest that domestication provides animals with plentiful food but may reduce their fermentation efficiency by reshaping the fecal microbiota, especially with regards to the efficiency of cellulose and hemicellulose degradation.

Generally, the fecal microbiota has higher diversity than the rumen microbiota (Meale et al., 2016), which may be due to the fact that the hindgut has to decompose the recalcitrant substances, as the digestible starch, fat, and protein have been almost digested in rumen. The residual substances were often recalcitrant to degrade for the rumen microbiota. Thus, the hindgut has to develop a more complex microbial structure to cope with the recalcitrant substances, for instance, higher microbial diversity, higher *Firmicutes*, lower *Bacteroidetes*, while the rumen microbiota has a lower diversity, predominant *Bacteroidetes* and lower *Firmicutes* (Meale et al., 2016; Andrade et al., 2020). Higher *Firmicutes* was often associated with degradation cellulose and hemicellulose and higher SCFAs content (Mao et al., 2012). Higher *Firmicutes/Bacteroidetes* was often considered as a biomarker of efficient energy extraction from diet (Turnbaugh et al., 2008). Thus, hindgut microbiota may focus on the degradation of recalcitrant substances compared to rumen microbiota.

Generally, the concentration of SCFAs was positively correlated with their gut microbial diversity and activity of fiber degradation (Li et al., 2018), represented a high energy harvest in plateau pikas and yaks (Zhang Z. et al., 2016; Li et al., 2018). The SCFAs verified the microbial activity in bears (Schwab et al., 2009). Yaks had a higher SCFAs content compared to cattle, which represented more effective metabolic pathways for hydrogen consumption and lower methane emission (Huang et al., 2012, 2016; Zhang Z. et al., 2016). In our study, the concentrations of six types of SCFAs (acetic, propionic, n-butyric, i-butyric, n-valeric, and i-valeric acid) were highest in wild yaks and lowest in domestic yaks (Figures 5A–F). Likewise, wild yaks have the largest body size, and domestic yaks have the smallest body size (Supplementary Tables 5–7). These scenes indicated that domestication might cause potential negative effects to the producing of SCFAs via dietary fermentation in yaks. The concentration of SCFAs corresponds to the reads of the GH family, which is responsible for encoding cellulases and endohemicellulases, and corresponds well with a previous report indicating that cellulolytic activity is positively correlated with the SCFA concentrations (Li et al., 2018). Therefore, the SCFA concentrations may further confirme that the cellulolytic activity in wild yaks was higher than that in domestic yaks, and that domestication weakened the digestive capacity in yaks.

The connections between the gut microbiome and SCFAs were consistent with the previous studies. For instance, the *Prevotella* spp. and *Ruminococcus* spp., which produce acetic acid from pyruvate via acetyl-CoA (Louis et al., 2014), were higher in wild yaks (Figure 3A,C). Correspondingly, the concentration of acetic acid was higher in wild yaks (Figure 5A). Likewise, the synchronization was also observed between propionic acid and the gut microbial producer. *Coprococcus* spp., which produce propionic acid and butyric acid (Louis et al., 2014), were higher in wild yaks (Figure 3A).

The domestication of animals is a long-term event; people preferred animals with less aggressiveness and more tameness rather than large-body in the early stage of domestication, as large-body individuals may hurt the people, especially in the domestication of large animals (Diamond, 2002; Jensen, 2014), this scene was also observed in yaks (Qiu et al., 2015). Modern genetic methods have been used to breed efficient and economical commercial lines of domesticated species only in recent years, while many local species have been domesticated by people living in less developed area (Zeder, 2015), including yaks (Qiu et al., 2015). The domestic yak is still in the early phase of domestication, without the experience of modern genetic breeding methods (Qiu et al., 2015). These locally domesticated animals in less developed area often exhibit bad growth performance with small body size compared to modern commercial lines, for example Landrace sows have a stronger capacity for fiber degradation and produce more SCFAs in the gut than Meishan and Jinhua sows (two local pigs in China) (Li et al., 2009; Ai et al., 2013).

The gut microbiota of hybrid offspring is different from their parents, as both the male and female parents gene expression in the hybrid offspring (Zhipeng et al., 2016). In this study, we collected three types of fecal samples in the same environment to exclude the influence of environment. Here, hybrids were considered as intermediaries to explore the influence of domestication. In composition, diversity, functional pathway, network, enzyme system and SCFAs, the hybrids showed an intermediate type, implied that they may be influenced by both male parent and female parent, meanwhile it implied that stable characters have been fixed during domestication. Hybridization may partly recover the composition and function of gut microbiota and promote the energy harvest capacity of the host (Benis et al., 2015; Zhipeng et al., 2016). These results corresponded well with previous studies that indicated the gut microbiota experienced vertical transmission with host genetics during the process of hybridization and was affected by the heritable character of parents (Wang et al., 2015; Zhipeng et al., 2016).

## Conclusion

In summary, our study provides novel insights into the effects of domestication and hybridization on the fecal microbiota, may further enlighten other researchers to investigate the role of fecal microbiota in livestock growth and development, and may provide a promising way to improve the growth performance of livestock by revising the fecal microbiota.

## Data Availability Statement

The 16S rDNA as well as the whole-metagenome data in this study can be freely retrieved from the NCBI Sequence Read Archive with project accession Nos. PRJNA528194 and PRJNA529943, respectively.

## Ethics Statement

The animal study was reviewed and approved by the Animal Ethics Committee of Northwest Plateau Institute of Biology, Chinese Academy of Sciences. Written informed consent was obtained from the owners for the participation of their animals in this study.

## Author Contributions

YZ and XZ designed the research. HF, CL, LZ, CF, and WL collected the samples. JL provided the sampling site and live specimen. CL and CF measured the content of SCFAs. HF, SJ, LZ, and CF analyzed the data. HF and CL wrote the draft of manuscript. YZ, SJ, and LZ revised the final manuscript. All authors contributed to the article and approved the submitted version.

## Conflict of Interest

The authors declare that the research was conducted in the absence of any commercial or financial relationships that could be construed as a potential conflict of interest.

## Footnotes

1 http://drive5.com/uparse/

2 http://greengenes.lbl.gov/cgi-bin/nph-index.cgi

3 http://soap.genomics.org.cn/soapaligner.html

4 http://csbl.bmb.uga.edu/dbCAN/download/CAZyDB.03172015.fa

5 ftp://ftp.ncbi.nlm.nih.gov/genomes/all/

6 http://www.bork.embl.de/pal2nal/

## Supplementary Material

Supplementary Figure 1 Composition of the fecal microbiota of the three types of yaks at the phylum level, the letters “D,” “H,” and “W” represent domestic, half-blood, and wild yaks, respectively. **(A)** Bray-Curtis dissimilarity of gut microbial communities among the three types of yaks at phylum level, Ellipses with 95% confidence interval around the centroid of each group are displayed in PCoA, the phyla which have significant correlation with the ordination in PCoA are displayed using the arrows (permutation test, *p* < 0.01), with the length of the arrow representing the goodness of fit statistic, squared correlation coefficient. **(B)** Heatmap of phylum based on the relative abundance of gut microbiota among the three types of yak, and complete linkage clustering was used. **(C)** Relative abundance of the gut microbiota indicating the significantly discrepancies among the three types of yak at the phylum level across 29 samples.

Supplementary Figure 2 The linear discriminant analysis effect size (LEfSe) shows the significantly different taxa of the gut microbiota between the different groups at phylum level (*LDA* scores > 2.0, *p* < 0.05); each line of the heatmap corresponds to the significantly different taxonomic result of each line in LEfSe on the left; the letters “D”, “H” and “W” represent domestic, half-blood, and wild yaks, respectively.

Supplementary Table 1 Nutrition composition of diet in Datong Breeding Farm (Zhang et al., 2014; Zhou et al., 2015).

Supplementary Table 2 Average relative abundance of gut microbiota across the three types of yaks at the phylum level.

Supplementary Table 3 Statistical differences of phyla and top 30 genera among three types of yaks.

Supplementary Table 4 The proportion of bacteria encoding the GH families (GH10, GH28, GH53, and GH5) across the three types of yaks.

Supplementary Table 5 Measurements of the Kunlun type of wild yak (Jiye et al., 2005).

Supplementary Table 6 Variation in body weight with season and group (Hu, 2001). G1 (May 2000), G2 (October 2000), and G3 (April 2000). WY (wild yak), TZ (domestic yak living in Tianzhu, Gansu Province), and DT (Daton g yak, F1 generation of wild yak × domestic yak).

Supplementary Table 7 Live weight and linear body measurements of domesticated and crossbred yaks at 6 and 18 months of age (Jialin et al., 1998).
