# Supplementary figures and images for: Domestication Shapes the Community Structure and Functional Metagenomic Content of the Yak Fecal Microbiota
Source: Front Microbiol. 2021 Mar 31;12:594075. doi: 10.3389/fmicb.2021.594075 (PMC8059439; doi:10.3389/fmicb.2021.594075)

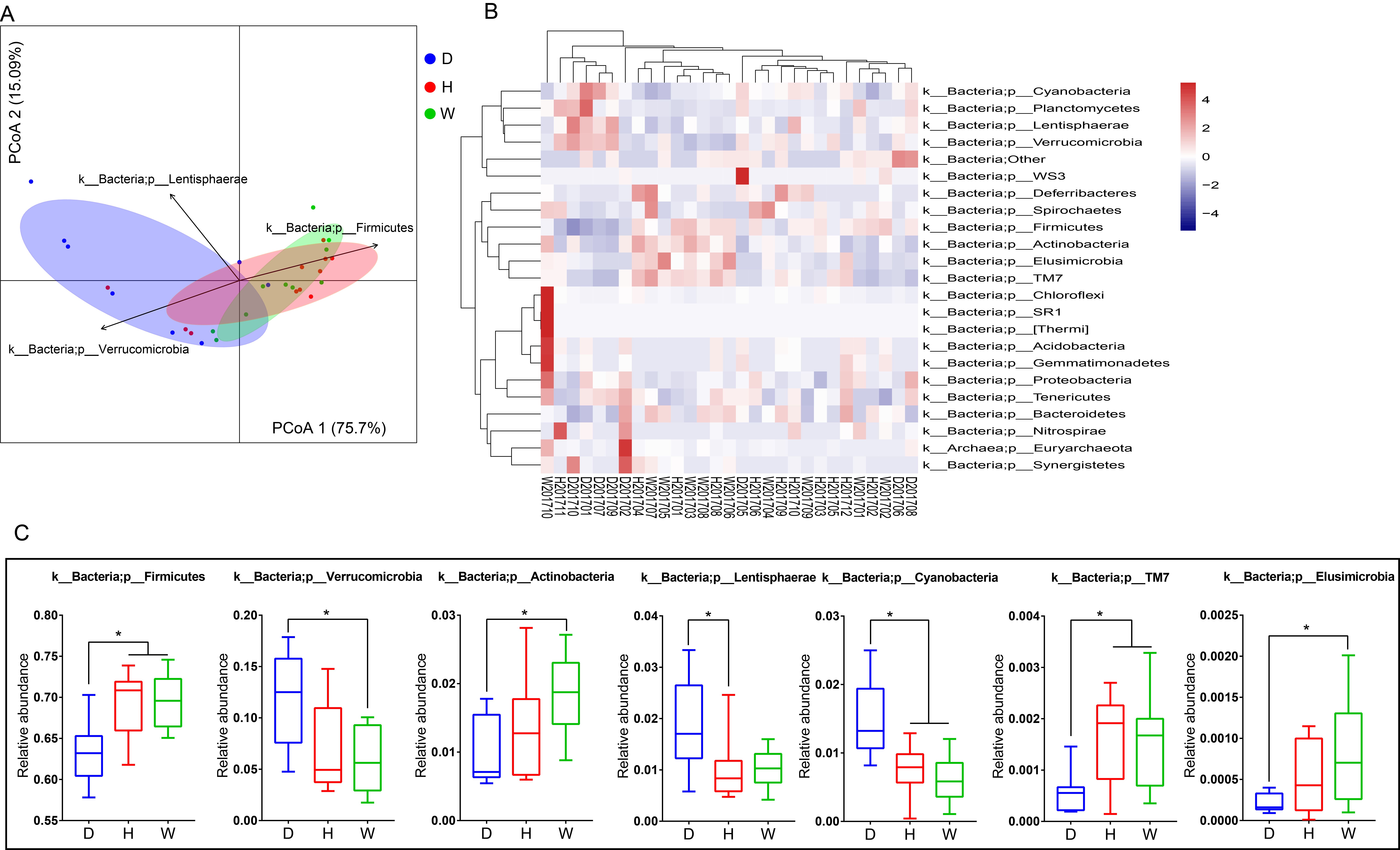

Supplement: Supplementary Figure 1 — Composition of the fecal microbiota of the three types of yaks at the phylum level, the letters “D,” “H,” and “W” represent domestic, half-blood, and wild yaks, respectively. (A) Bray-Curtis dissimilarity of gut microbial communities among the three types of yaks at phylum level, Ellipses with 95% confidence interval around the centroid of each group are displayed in PCoA, the phyla which have significant correlation with the ordination in PCoA are displayed using the arrows (permutation test, p < 0.01), with the length of the arrow representing the goodness of fit statistic, squared correlation coefficient. (B) Heatmap of phylum based on the relative abundance of gut microbiota among the three types of yak, and complete linkage clustering was used. (C) Relative abundance of the gut microbiota indicating the significantly discrepancies among the three types of yak at the phylum level across 29 samples. [file Image_1.TIF]

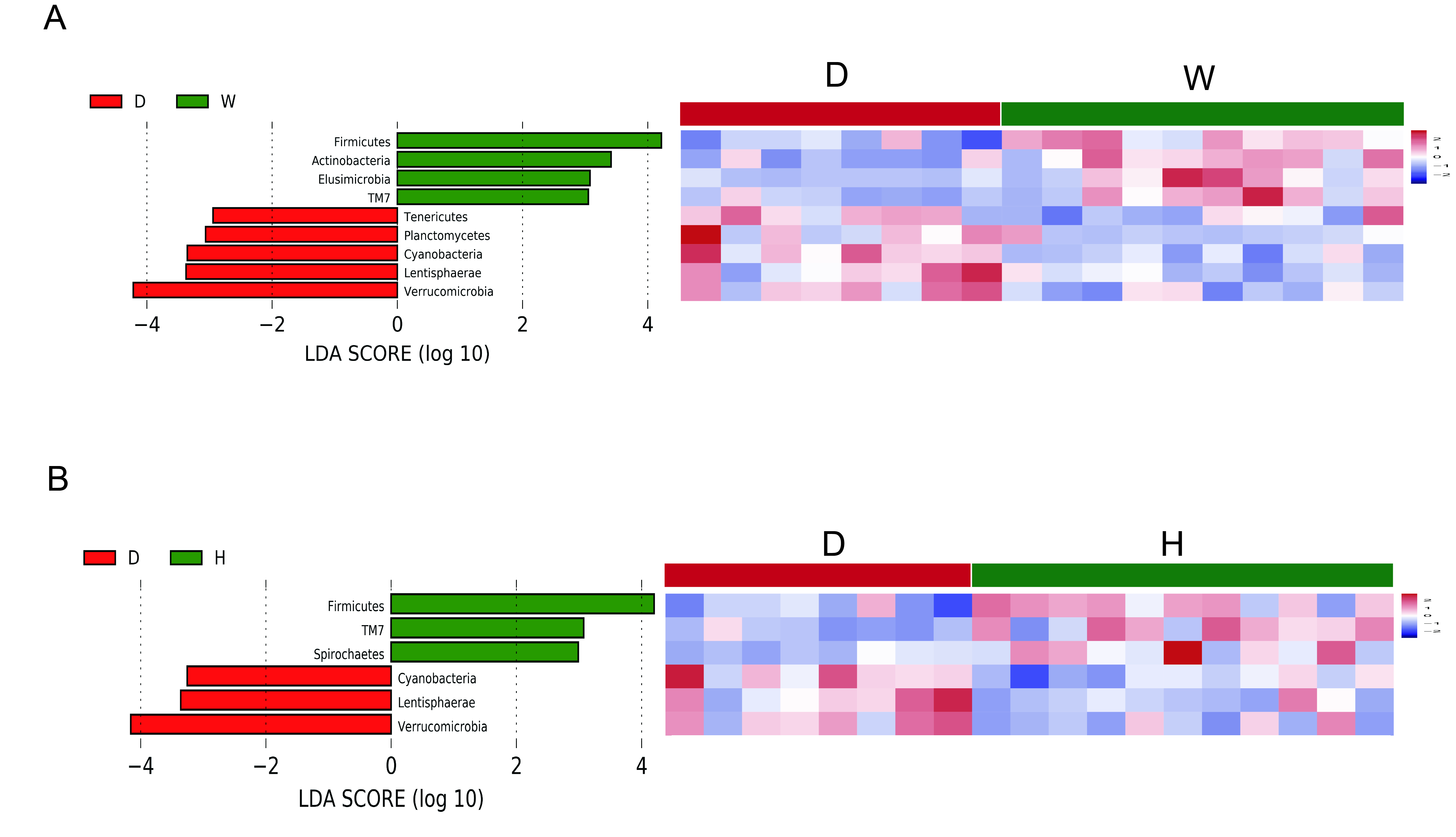

Supplement: Supplementary Figure 2 — The linear discriminant analysis effect size (LEfSe) shows the significantly different taxa of the gut microbiota between the different groups at phylum level (LDA scores > 2.0, p < 0.05); each line of the heatmap corresponds to the significantly different taxonomic result of each line in LEfSe on the left; the letters “D”, “H” and “W” represent domestic, half-blood, and wild yaks, respectively. [file Image_2.TIF]
