# Supplementary material for: Domestication Shapes the Community Structure and Functional Metagenomic Content of the Yak Fecal Microbiota
Source: Front Microbiol. 2021 Mar 31;12:594075. doi: 10.3389/fmicb.2021.594075 (PMC8059439; doi:10.3389/fmicb.2021.594075)
Supplement: Supplementary Table 1 — Nutrition composition of diet in Datong Breeding Farm (Zhang et al., 2014; Zhou et al., 2015). [file Data_Sheet_1.doc]

**Table S1:** Nutrition composition of diet in Datong Breeding Farm (Zhang et al., 2014;Zhou et al., 2015).

| Items | Content(%) | |
| --- | --- | --- |
| Crude protein | | 8.24 |
| Crude fat | | 2.30 |
| Neutral detergent fiber | | 49.86 |
| Acid detergent fiber | | 35.89 |
| Ca | | 1.35 |
| Total P | | 0.78 |

Zhang, H.B., Wang, Z.S., Peng, Q.H., Tan, C., and Zou, H.W. (2014). Effects of different levels of protein supplementary diet on gene expressions related to intramuscular deposition in early-weaned yaks. *Animal Science Journal* 85**,** 411-419.

Zhou, A., Wang, W., Wang, Z., Zou, H., Peng, Q., Feng, Y., He, X., Yin, M., and La, H. (2015). Effect of Different Protein Levels of Supplementary Diets on Performance and Gastrointestinal Development in Early-Weaned Yak Calves. *Chinese Journal of Animal Nutrition* 27**,** 918-925.

**Table S3:** Statistical differences of phyla and top 30 genera among three types of yaks.

| Gut microbial taxa | | Relative abundance | | | Kruskal-Wallis |  |
| --- | --- | --- | --- | --- | --- | --- |
|  |  | D | H | W | χ² | corrected  *P*-value |
|  | k__Bacteria;p__Firmicutes | 0.63230 | 0.69289 | 0.69578 | 10.062 | 0.007 |
|  | k__Bacteria;p__Verrucomicrobia | 0.11931 | 0.06635 | 0.05745 | 8.497 | 0.014 |
|  | k__Bacteria;p__Actinobacteria | 0.00970 | 0.01364 | 0.01857 | 7.362 | 0.025 |
| Phyla | k__Bacteria;p__Lentisphaerae | 0.01869 | 0.00981 | 0.01026 | 7.143 | 0.028 |
|  | k__Bacteria;p__Cyanobacteria | 0.01473 | 0.00788 | 0.00611 | 13.473 | 0.001 |
|  | k__Bacteria;p__TM7 | 0.00057 | 0.00166 | 0.00153 | 9.391 | 0.009 |
|  | k__Bacteria;p__Elusimicrobia | 0.00021 | 0.00052 | 0.00085 | 6.443 | 0.040 |
|  |  |  |  |  |  |  |
|  | g__Akkermansia | 0.10036 | 0.05758 | 0.04751 | 7.867 | 0.020 |
|  | o__Bacteroidales;f__;g__ | 0.04444 | 0.05546 | 0.05673 | 10.378 | 0.006 |
|  | g__Oscillospira | 0.02456 | 0.02977 | 0.03043 | 9.769 | 0.008 |
|  | f__Lachnospiraceae;g__ | 0.01827 | 0.03006 | 0.02760 | 13.342 | 0.001 |
| Genera | f__RF16;g__ | 0.00879 | 0.01347 | 0.01385 | 11.211 | 0.004 |
|  | f__Victivallaceae;g__ | 0.01784 | 0.00960 | 0.00974 | 7.143 | 0.028 |
|  | f__Coriobacteriaceae;g__ | 0.00690 | 0.01110 | 0.01472 | 7.451 | 0.024 |
|  | f__RFP12;g__ | 0.01736 | 0.00824 | 0.00939 | 11.061 | 0.004 |
|  | o__YS2;f__;g__ | 0.01473 | 0.00788 | 0.00609 | 13.473 | 0.001 |
|  | g__Dorea | 0.00504 | 0.00839 | 0.00763 | 13.110 | 0.002 |

**Table S5:** Measurements of the Kunlun type of wild yak (He et al 2005).

| Age(month) | No. | Height(cm) | Length(cm) | Heart girth(mm) | Cannon bone circumference(cm) | Weight(kg) |
| --- | --- | --- | --- | --- | --- | --- |
| 3 | 10 | 76.7±7.8 | 71.8±4.8 | 81.7±4.5 | 10.4±0.5 |  |
| 4 | 10 | 80.6±6.3 | 78.0±9.8 | 86.8±5.2 | 11.4±0.4 | 39.1±3.5 |
| 8 | 5 | 88.0±2.3 | 92.1±3.6 | 109.9±7.5 | 12.6±0.7 | 53.5±15.9 |
| 18 | 4 | 102.5±2.6 | 110.5±2.6 | 131.0±6.2 | 13.8±0.5 | 133.9±14.7 |
| 20 | 4 | 110.8±3.3 | 116.5±3.9 | 138.9±8.6 | 14.4±0.9 | 152.6±26.8 |
| 24 | 3 | 140.0±2.6 | 143.3±2.5 | 187.3±16.2 | 18.8±1.0 | 399.0±46.7 |

HeXiaolin, WuKexuan, Lahuan, LiJiye, HeXiaolin, WuKexuan *et al* (2005). Growth and Development of The KunlunType of Wild Yak. *China Herbivores***:** 227-228.

**Table S6:** Variation in body weight with season and group (HuJ 2001). G1 (May 2000), G2 (October 2000), G3 (April). WY (wild yak), TZ (domestic yak living in Tianzhu, Gansu, Province), and DT (F1 generation of wild yak × domestic yak).

| Age | Sample time | WY(male)  n=4 | TZ(male)  n=5~15 | DT(female)  n=18~21 | TZ(female)  n=3~13 |
| --- | --- | --- | --- | --- | --- |
|  | G1 | 70.88±9.75 | 65.30±13.48 | 85.69±13.08 | 60.81±11.92 |
| 1 | G2 | 132.40±15.42 | 112.00±20.99 | 116.47±10.03 | 144.83±29.33 |
|  | G3 | 152.63±26.80 | 105.04±9.90 | 129.50±20.44 | 83.60±9.05 |
|  | G1 |  | 158.83±46.26 | 138.30±23.80 | 101.68±35.31 |
| 2 | G2 |  | 163.17±20.33 | 164.97±22.93 | 122.91±16.32 |
|  | G3 |  | 146.44±20.69 | 180.98±40.65 | 133.65±24.87 |

HuJ (2001). Study on the biological characteristics and genetic diversities of wild yak and domestic yak and their hybrid.

**Table S7:** Live weight and linear body measurements of domesticated and crossbred yaks at 6 and 18 months of age (Jialin et al., 1998).

|  | Number | Age  (months) | Height at withers  (mm) | Body  length  (mm) | Live weight  (kg) |
| --- | --- | --- | --- | --- | --- |
|  |  |  | Mean s.e | Mean s.e | Mean s.e |
| Crossbred yak | 7 | 6 | 844.3 56.2 | 870.7 52.3 | 74.71 10.41 |
| Domestic yak | 7 | 6 | 793.6 34.2 | 820.0 45.5 | 59.83 10.23 |
| Difference |  |  | 50.7 | 50.7 | 14.88 |
| Crossbred yak | 6 | 18 | 1031.0 24.0 | 1085.0 46.8 | 150.50 56.07 |
| Domestic yak | 6 | 18 | 1085.0 34.6 | 1031.8 28.0 | 117.73 17.42 |
| Difference |  |  | 26.7 | 53.2 | 32.77 |

Jialin, B., Mingqiang, W., Zhonglin, L., and Chesworth, J.M. (1998). Meat production from crossbred and domestic yaks in China. *Animal Science* 66**,** 465-469.
